# Falling Out of Time: Enhanced Memory for Scenes Presented at Behaviorally Irrelevant Points in Time in Posttraumatic Stress Disorder (PTSD)

**DOI:** 10.1371/journal.pone.0042502

**Published:** 2012-07-31

**Authors:** Einat Levy-Gigi, Szabolcs Kéri

**Affiliations:** 1 Rutgers University, Center for Molecular and Behavioral Neuroscience, Newark, New Jersey, United States of America; 2 National Psychiatry Center, Budapest, Hungary; 3 University of Szeged, Faculty of Medicine, Department of Physiology, Szeged, Hungary; Max Planck Institute of Psychiatry, Germany

## Abstract

Spontaneous encoding of the visual environment depends on the behavioral relevance of the task performed simultaneously. If participants identify target letters or auditory tones while viewing a series of briefly presented natural and urban scenes, they demonstrate effective scene recognition only when a target, but not a behaviorally irrelevant distractor, appears together with the scene. Here, we show that individuals with posttraumatic stress disorder (PTSD), who witnessed the red sludge disaster in Hungary, show the opposite pattern of performance: enhanced recognition of scenes presented together with distractors and deficient recognition of scenes presented with targets. The recognition of trauma-related and neutral scenes was not different in individuals with PTSD. We found a positive correlation between memory for scenes presented with auditory distractors and re-experiencing symptoms (memory intrusions and flashbacks). These results suggest that abnormal encoding of visual scenes at behaviorally irrelevant events might be associated with intrusive experiences by disrupting the flow of time.

## Introduction

Posttraumatic stress disorder (PTSD) may develop after exposure to psychological trauma that threatens basic security and exceeds coping abilities [1]. PTSD symptoms include intrusive mental contents (unwanted memories, flashbacks, and nightmares), avoidance of thoughts and cues related to trauma, emotional numbing, and increased vigilance. Although memory is usually impaired in PTSD, the coexistence of avoidance and intrusions highlights the Janus-face of these symptoms. There are deficits in voluntary control (e.g., inability to recall basic details of events) and enhancement of spontaneous memories (unintended re-experiencing) [2]–[8].

Brewin [9] defined three aspects of memory that predict progression of PTSD and may have a causal role in the development of symptoms: integration of trauma with identity, disorganized contextual memories, and sensation-based memories/flashbacks. Disorders of contextualization refer to a lessened ability of patients to recall coherent and integrated narratives of trauma-related autobiographical memories. Instead, patients often experience intrusive and emotion-laden fragments of traumatic events (flashbacks), which are vivid, stereotyped, and sensual images [9]. Despite a growing amount of evidence suggesting that memory dysfunctions are critical in PTSD, basic mechanisms of visual memory for complex information and its modulation by behavioral context are unknown.

**Figure 1:**
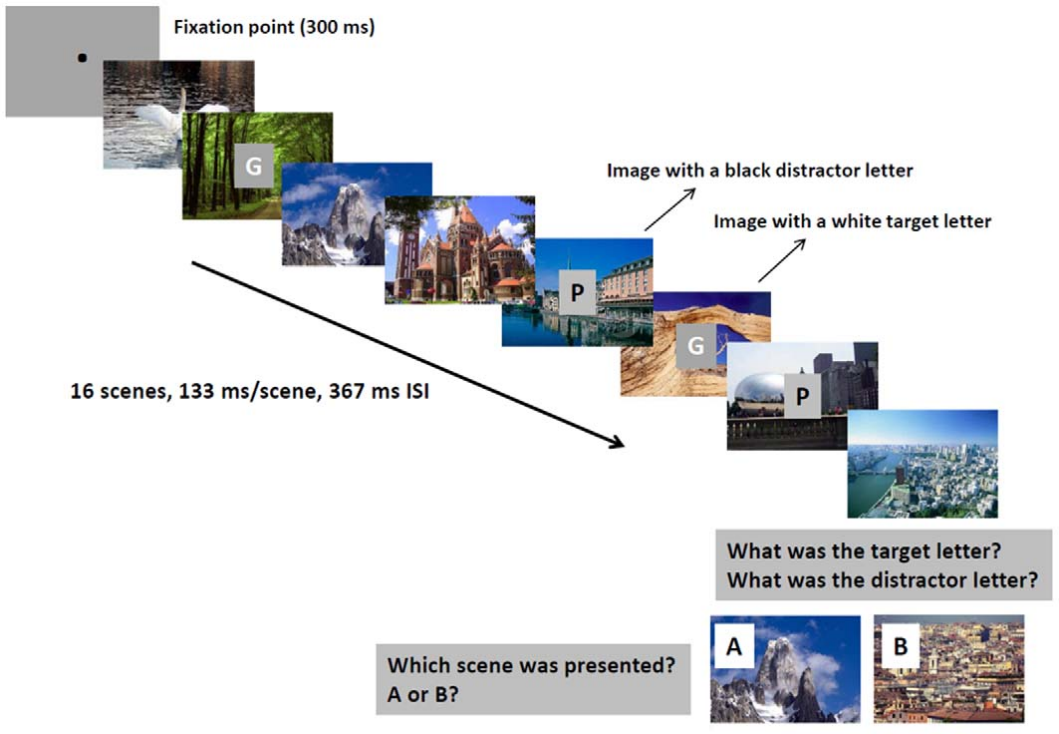
Illustration of a scene sequence. Participants were asked to press a key to start the sequence. Out of the 16 scenes presented, two contained white target letters that should be remembered and two contained black distractor letters that should be omitted (in the figure, only eight scenes are shown). Following the sequence, participants were first requested to type the target and distractor letter and then to decide which of two scenes was presented in the sequence (“A” or “B”). In the auditory condition, target and distractor tones were presented instead of letters. ISI – inter-stimulus interval.

Humans have a tremendous capacity to perceive visual scenes but recognition memory for them is often weak [10]–[15]. However, if investigators use a rapid serial visual presentation paradigm and expose scenes at behaviorally relevant points in time (i.e., together with a target stimulus), recognition memory is better relative to trials when scenes appear at behaviorally irrelevant points in time (i.e., together with distractors) [16]. Greater attention allocation may explain this enhanced memory, which facilitates not only the processing of the behaviorally relevant target, but also its background, the scene [17]. In the current study, we used this paradigm to investigate scene perception and memory (Figure 1).

We studied whether individuals with PTSD are able to use behaviorally relevant events (detecting target letters and auditory tones) to increase recognition memory for background scenes. We predicted that individuals with PTSD exhibit a reduced capacity to develop memory traces for target letters/tones and background scenes at behaviorally relevant points in time because of their dysfunction of attentional control [18]. We also tested whether individuals with PTSD are able to create memory representations of trauma-related scenes in a rapid serial presentation paradigm. We hypothesized that attention is enhanced for trauma-related scenes relative to neutral scenes in PTSD. Therefore, the prediction was that individuals with PTSD show better recognition performance for trauma-related scenes as compared to controls.

## Materials and Methods

### Participants

Forty individuals with PTSD, including seven late-onset cases when symptoms appeared more than six months after the traumatic event, and 40 control volunteers without PTSD participated in the study. On October 4, 2010, a damn of a sludge reservoir owned by an aluminum company had ruptured, and a mixture of toxic red sludge inundated the settlements of Kolontár, Devecser, and Somlóvásárhely. It was Hungary's largest ecological disaster (http://redsludge.bm.hu/). Individuals with PTSD and controls lived in the red sludge-affected area and personally witnessed the disaster. Volunteers were recruited via local psychiatric units, general practitioners, and self-help organizations. Exclusion criteria included history of other psychiatric and neurological disorders, including former PTSD related to other traumatic events and psychoactive substance misuse. All participants were offered the option to be referred to treatment regardless of their involvement in the study. Participants did not receive monetary compensation. The assessment approximately took two hours.

We conducted the assessments prior to therapy. Therefore, participants did not receive medications or psychotherapy at the time of testing. We administered the Structured Clinical Interview for DSM-IV axis I disorders (SCID-CV) [19], the Trauma and Life Events Self-report Inventory (TLESI) [20], the Clinician-Administered PTSD Scale (CAPS) [21], the Hamilton Depression Rating Scale (HAM-D) [22], and the Wechsler Abbreviated Scale of Intelligence (WASI) [23]. The scales were administered by trained clinicians who were not aware of the data obtained from the experiments. Table 1 depicts the demographic and clinical characteristics.

**Table 1:** Demographic and clinical characteristics of the participants.

	PTSD (n = 40, 11 male, 29 female)	Controls (n = 40; 11 male, 29 female)
Age (years)	41.3 (7.6)	41.9 (8.7)
Education (years)	10.9 (5.4)	11.1 (6.1)
IQ	102.1 (11.5)	103.0 (12.0)
TLSI*	5.1 (2.1)	3.1 (1.9)
HAM-D**	15.0 (6.0)	8.4 (3.4)
Duration of symptoms (months)	4.3 (1.8)	-
CAPS		
Re-experiencing	19.4 (7.0)	-
Hyperarousal	31.0 (6.5)	-
Avoidance	23.7 (6.7)	-

Data are mean (standard deviation). TLSI - Trauma and Life Events Self-report Inventory (mean number of traumatic and adverse life events), HAM-D - Hamilton Depression Rating Scale, CAPS - Clinician-Administered PTSD Scale,

* t(78) = −4.39, p<0.001;

** t(78) = −6.10, p<0.001.

### Ethics statement

The study was approved by the institutional ethics committee (University of Szeged, No. 2697/2010) and was done in accordance with the Declaration of Helsinki. After full description of the study, all participants gave written informed consent.

### Stimuli

We used a VP2765-LED-27″ monitor for stimulus presentation (ViewSonic, Walnut, CA; refresh rate: 60 Hz; resolution: 1920×1080 pixel; viewing distance: 50 cm; output luminance: 65 cd/m^2^). Stimuli were photographs (size: 28 degrees of visual angle) from the LabelMe Natural and Urban Scenes database [24] (http://cvcl.mit.edu/database.htm) and a previously used stimulus set [14]. We also made 250 photographs of the red sludge disaster, which served as trauma-related stimuli. All stimuli were adjusted to meet the properties of the LabelMe database [24]. Ten trauma-exposed individuals rated each stimulus for emotional valence and trauma-related features on a five-level Likert scale (1 – emotionally neutral, trauma unrelated; 5 – emotionally laden, trauma-related). In the neutral condition, we included only scenes with a score of 1 point, whereas in the trauma-related condition we used scenes that received 4–5 points.

### Procedure

We tested participants on three different tasks (described below as Experiments 1–3). The order of the tasks was counterbalanced across participants. The experiments were separated by breaks in order to avoid fatigue.

#### Experiment 1: Neutral scenes with visual target

We used the modified method of Lin et al. [16]. Each trial included a rapid serial presentation stream of 16 scenes (exposure time: 133 msec/scene, inter-stimulus interval: 367 msec). This presentation rate is slow enough to avoid attentional blink [16]. A gray square (size: 1 degree of visual angle) appeared in the center of some scenes. The square contained white target or black distractor letters (type: Calibri; font size: 20) (Figure 1). Target and distractor letters appeared in the center of two-two non-consecutive scenes out of the total 16 scenes. The remaining 12 scenes contained neither target nor distractor letters. Participants were requested to remember target letters and to ignore distractor letters. Following each trial, we first asked the participants to type the target and the distractor letter. After the letter recall phase, two test scenes (“A” and “B”) were exposed for 3000 msec. One of these scenes was from the sequence (serial position: 6–14), whereas the other scene was new. We asked the participants to choose which of the scenes appeared in the sequence by pressing key “A” or “B” on the computer keyboard (Figure 1). The test stimulus could be a scene without a letter, with a target letter, or with a distractor letter in the sequence. We applied 300 intermixed trials (10 blocks of 30 trials) separated by breaks. Before the test, we administered a training session of 30 trials for each participant, but they were not familiarized with the test scenes.

#### Experiment 2: Neutral scenes with auditory target

Stimulus presentation was similar to that described in Experiment 1 with the exception that each scene was paired with a brief auditory tone (duration: 50 msec). No letters were presented. The frequency of baseline tones was 260 Hz (40 dB). Baseline tones were paired with 12 out of the 16 scenes. Target tones were presented together with two non-consecutive scenes. The frequency of the target tones was either 130 Hz (low pitch) or 520 Hz (high pitch). Two scenes appeared together with distractor tones, which were louder than the baseline tones (60 dB). Participants were asked to ignore the distractor tones. Following the trial, the task was to discriminate the pitch of the target tones as either lower or higher than the baseline tones. Participants responded by pressing two different keys (“A” for high, “B” for low) on the computer keyboard. After the tone discrimination, there was a scene recognition task as described in Experiment 1. The number of trials was the same as in Experiment 1.

#### Experiment 3: Neutral vs. trauma-related scenes

We presented sequences of scenes and tested recognition performance as described in Experiment 1. We did not use letters or tones. Two of the 16 scenes were trauma-related, whereas 14 stimuli were neutral. There were 200 intermixed trials (10 blocks of 20 trials; 100 trials testing the recognition of trauma-related scenes and 100 trials for neutral scenes).

### Data analysis

We performed data analysis using STATISTICA 9 software (StatSoft Inc., Tulsa). The primary dependent measure was scene recognition performance (percentage of correct judgments). The normal distribution of the data was evaluated with Kolmogorov-Smirnov tests, whereas the homogeneity of variance was explored with Levene's tests. We used repeated measures analyses of variance (ANOVAs) to examine the difference between individuals with PTSD and controls in the case of different stimuli. We applied Scheffé's tests for post hoc comparisons. Demographic data and test performances not included in ANOVAs (letter recall and tone discrimination) were compared with two-tailed *t* tests. Pearson's product moment correlation coefficients described the relationship between test performances and clinical scores. The level of statistical significance was alpha <0.05.

## Results

### Experiment 1

**Figure 2:**
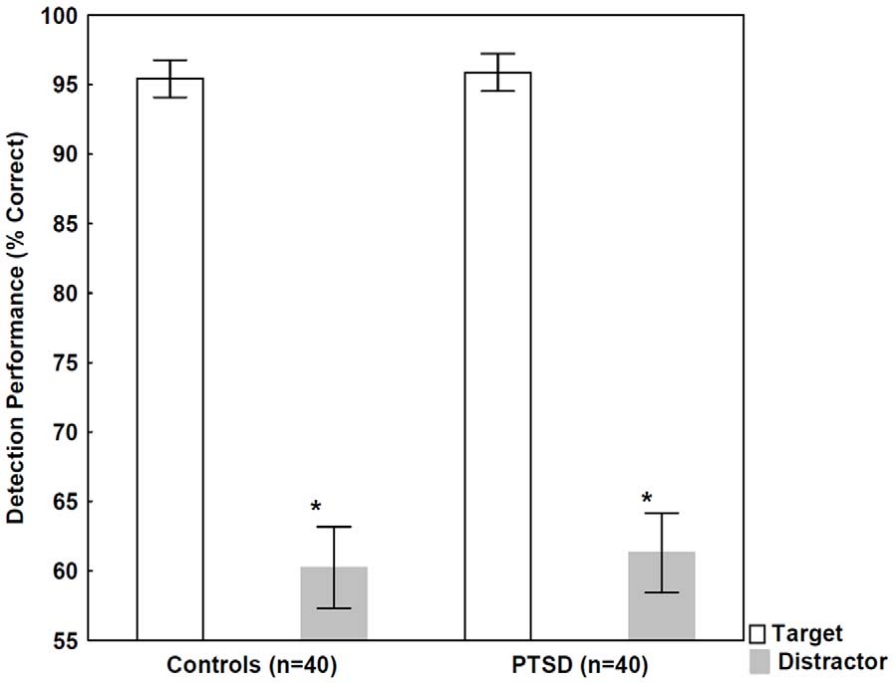
Recall performance for target and distractor letters in individuals with PTSD and controls. Error bars indicate 95% confidence intervals. The performance was significantly lower for distractors relative to targets (*p<0.001, *t* test), but the two groups did not differ.

The results from the three experiments are summarized in Table 2. Figure 2 depicts recall performances for target and distractor letters. There were no statistically significant differences between individuals with PTSD and controls in the case of target and distractor letters (*t* test, p>0.5). Participants exhibited a lower level of recall for distractor letters than for target letters (controls: t(78) = 22.10, p<0.0001; PTSD: t(78) = 22.15, p<0.001) (Figure 2).

**Table 2:** Behavioral results.

	Controls (n = 40)			PTSD (n = 40)		
	Mean	−95% CI	+95% CI	Mean	−95%CI	+95% CI
Target letter identification	95.4	94.1	96.8	95.8	94.5	97.2
Distractor letter identification	60.3	57.3	63.2	61.3	58.4	64.1
Scenes with no letters	50.6	49.4	51.8	50.7	49.4	51.9
Scenes with target letters*	68.2	66.1	70.3	54.5	52.1	56.9
Scenes with distractor letters*	50.5	49.4	51.6	61.1	58.4	64.2
Scenes with baseline tones	50.5	49.0	51.9	49.4	47.5	51.3
Scenes with target tones*	63.2	61.1	65.3	53.7	51.4	56.0
Scenes with distractor tones*	51.4	49.7	53.1	58.3	55.6	61.1
Trauma-related scenes	-	-	-	51.5	48.9	54.2
Neutral scenes with no letters or tones	-	-	-	52.9	50.0	55.8

Mean letter identification and scene recognition performances (% correct) and 95% confidence intervals (CI) in controls and individuals with posttraumatic stress disorder (PTSD).

* Significant differences, Scheffé's tests.

**Figure 3:**
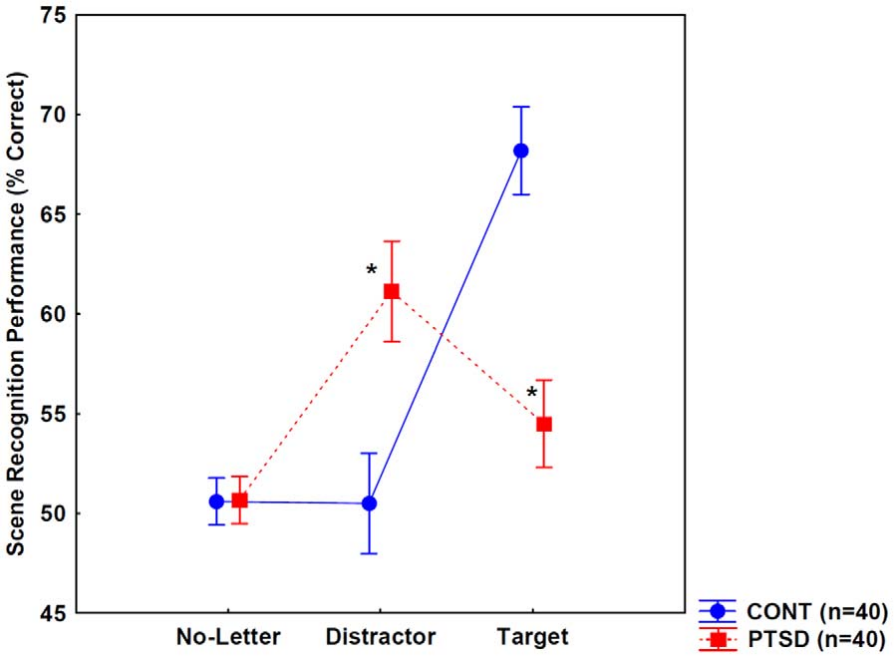
Scene recognition performance when stimuli were presented without letters, with distractor letters, and with target letters. Individuals with posttraumatic stress disorder (PTSD) achieved higher recognition performance for scenes with distractors relative to controls (CONT), whereas the opposite was found for scenes with targets. Error bars indicate 95% confidence intervals. * p<0.001, Scheffé's tests.

Figure 3 depicts scene recognition performance from Experiment 1. We conducted a two-way ANOVA with group (PTSD vs. controls) as the between-subjects factor and stimulus type (no-letter, distractor letter, target letter) as the within-subjects factor. There was no significant main effect of group (p>0.3), whereas the effect of stimulus type was significant (F(2,156) = 66.54, p<0.001, η^2^ = 0.46). There was a significant interaction between group and stimulus type (F(2,156) = 88.26, p<0.001, η^2^ = 0.53).

Scheffé's tests conducted on the two-way interaction indicated similar performances in the PTSD and control group for scenes with no letters (p>0.5). Individuals with PTSD and controls did not differ from chance level in recognition performance (*t* test, p>0.2). In the case of scenes with distractor letters, individuals with PTSD achieved a higher recognition performance as compared to controls (p<0.001), whereas in the case of scenes with target letters, the opposite results were found, that is, controls displayed a higher level of recognition compared to individuals with PTSD (p<0.001) (Figure 3).

### Experiment 2

**Figure 4:**
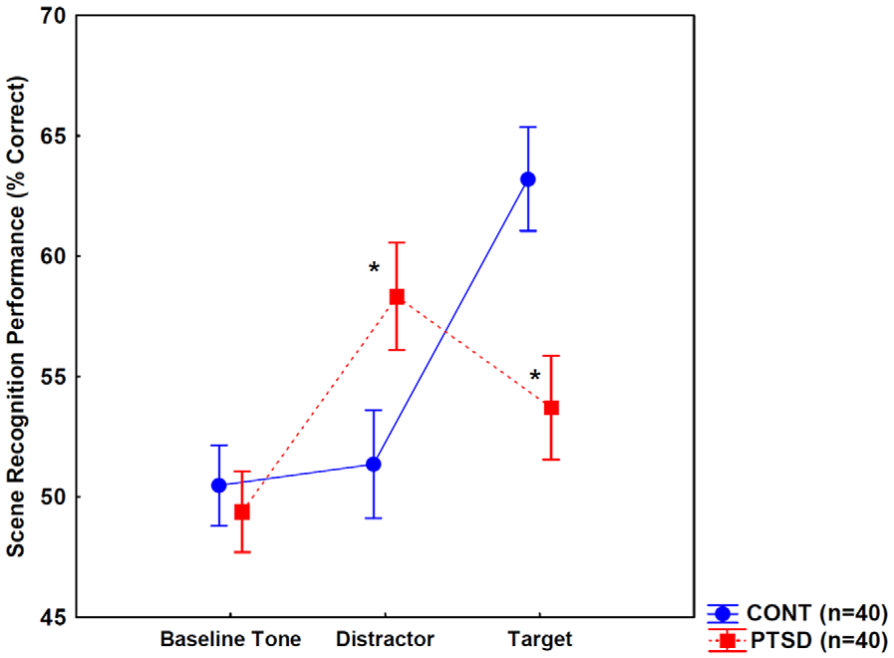
Scene recognition performance when stimuli were presented with baseline, distractor, and target tones. Individuals with posttraumatic stress disorder (PTSD) achieved higher recognition performance for scenes with distractors relative to controls (CONT), whereas the opposite was found for scenes with targets. Error bars indicate 95% confidence intervals. * p<0.01, Scheffé's tests.

Pitch discrimination performance was similar in controls (96.2%, CI: 94.9–97.5) and individuals with PTSD (95.8%, CI: 94.4–97.2) (*t* test, p>0.5). The ANOVA design was the same as used in Experiment 1. Figure 4 depicts the results. The main effect of group (control vs. PTSD) was not significant (p>0.1), whereas the effect of stimulus type (scenes with baseline, target, and distractor tones) was significant (F(2,156) = 39.04, p<0.001, η^2^ = 0.33). Critically, the interaction between group and stimulus type was also significant (F(2,156) = 36.17, p<0.001, η^2^ = 0.32).

Scheffé's tests revealed that the two groups did not differ in the case of baseline tones (p>0.5). However, in the case of distractor tones, individuals with PTSD outperformed controls (p<0.01), and in the case of target tones, controls outperformed the PTSD group (p<0.001) (Figure 4). In controls, scene recognition performance did not differ from the chance level (50%) for baseline tones (*t* test, p>0.1).

### Experiment 3

**Figure 5:**
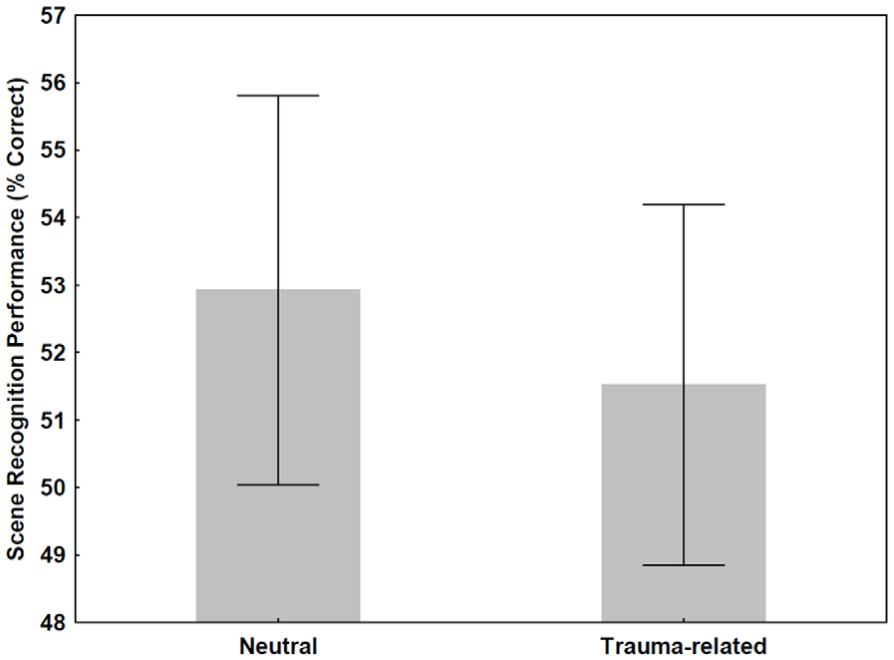
Scene recognition performance for neutral and trauma-related stimuli in PTSD. There was no significant difference between the recognition of these two types of scenes.

In the PTSD group, there was no significant difference between the recognition of neutral and trauma-related scenes (*t* test, p>0.4) (Figure 5).

### Gender differences and correlation with clinical symptoms

**Figure 6:**
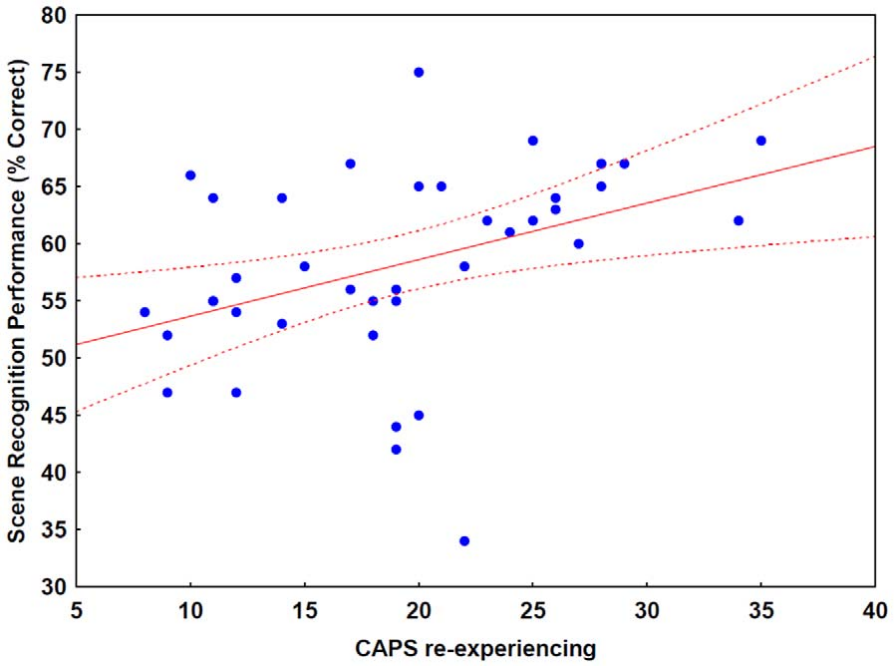
Correlation between recognition performance for scenes with auditory distractors and re-experiencing symptoms in PTSD. r = 0.41, p<0.05.

We found no differences between male and female participants in all three experiments (p>0.2). There were no significant correlations between CAPS avoidance, hyperarousal, and HAM-D scores and scene recognition performances in all three experiments (−0.2<r<0.2, p>0.1). A significant correlation was found between CAPS re-experiencing scores and scene recognition performance when distractor tones were applied in Experiment 2 (r = 0.41, p<0.05) (Figure 6), indicating that more severe re-experiencing symptoms were associated with a better recognition of scenes presented at irrelevant points in time.

## Discussion

Contrary to our hypothesis, individuals with PTSD showed intact performance on letter identification and tone discrimination tasks, suggesting that they were able to allocate attentional resources to target stimuli. In accordance with previous findings [16], [17], healthy controls showed a higher level of recognition in the case of background scenes presented with target stimuli. According to Swallow and Jiang [17], focused spatial attention on a target stimulus has a global impact on memory formation and facilitates the encoding of temporally coincident scenes [17]. It is not likely that arousal elicited by perceptual novelty can explain this effect [25], because recognition memory for the stimuli presented before or after the target was at chance [16].

Individuals with PTSD displayed impaired recognition of scenes presented with targets, suggesting a weakened effect of focused attention on the background. However, we found the opposite pattern of performance for distractors. Despite the fact that distractor omission was similarly successful in PTSD and controls (lower recall performance for distractors relative to targets), it did not lead to decreased encoding of temporally coincident scenes in the PTSD group. In the case of both visual and auditory distractors, individuals with PTSD outperformed controls in scene recognition. These findings suggest that individuals with PTSD do not have impairments in focal spatial attention *per se*, and they exhibit enhanced encoding of background information at behaviorally irrelevant points in time relative to that at behaviorally relevant events.

In the case of auditory distractors, scene recognition performance was associated with more severe re-experiencing symptoms in PTSD, including intrusive memories and flashback experiences. This relationship between scene memory and re-experiencing symptoms in the case of auditory distractors, but not in the case of visual distractors, is puzzling. A possible explanation is that auditory distractors elicited more attention than visual distractors, or audiovisual integration (sounds paired with scenes) more closely resembles real life scenarios than letters in front of scenes.

We found no evidence for greater encoding of trauma-related scenes relative to neutral images in PTSD. These results suggest that enhanced memory is not confined to trauma-related or emotionally arousing information [26]. Past studies have shown that, during rapid serial visual presentation, individuals with PTSD symptoms process trauma-related stimuli more rapidly and efficiently (i.e., “consuming less attentional resources”) [27], and these stimuli elicit enhanced frontotemporal and amygdala activation [28]. However, it does not necessarily mean enhanced conscious recognition. Our results might suggest that enhanced encoding of irrelevant peripheral information can be associated with overactive memory representations in PTSD. As an involuntary process, it may be one of the mechanisms in the development of intrusive memories and images [29], [30].

In the model of Brewin et al. [29], there are two types of memory representations. Contextual memory is abstract, verbal, extends to an attentional window as a part of prior knowledge. Sensation-based memory is situationally accessible, extends to the entire visual field, and supports immediate action [29]. Overactivity in the amygdala, linked to emotional arousal and fear in PTSD, may boost the activation of sensory cortical areas [31]–[33], which results in intrusive images. We described a possible mechanism for the emergence of sensation-based intrusive memories, which is based on the abnormal encoding of context at behaviorally irrelevant points in time. Contrary to the model of arousal-driven cortical activation and memory modulation [31]–[33], abnormal encoding of context (background scenes) is independent of trauma-related negative emotional contents when the symptoms of PTSD are present. Future studies are warranted to examine the relationship among abnormal encoding at behaviorally irrelevant events, stress, trauma exposure, and individual vulnerability. It is interesting, for example, whether participants in the control group could develop late-onset PTSD as well. In addition, future research should address whether this is a vulnerability factor (trait marker) or just present in individuals with current PTSD (state marker) and will disappear with effective treatment.

Why is visual information processing relevant to PTSD? Two pioneering studies demonstrated either increased [34] or decreased [35] activation in visual areas during the provocation of traumatic memories. Lanius et al. [36] found that, compared with controls, individuals with PTSD in a dissociative state showed more activation in the occipital lobe, which also displayed greater functional connectivity with other cortical areas [37]. Critically, visual activation in PTSD seems to be modulated by the level of information processing. Hendler et al. [38] found that responses to combat images evoked more activation in the visual cortex in people with PTSD than in non-PTSD controls, only when masked images were below recognition threshold. However, recognition threshold did not affect amygdala activation [38]. Although we did not use images below the detection threshold, short duration and rapid serial presentation are similar to the approach of Hendler et al. [38]. Evidence also suggests structural alterations of the visual cortex in PTSD [39]–[41]. These findings are particularly important in light of recent data, indicating that greater re-experiencing scores in PTSD correlated with reduced volume in the middle temporal and inferior occipital cortices [40].

Although several studies explored neuronal activity during rapid serial visual presentation [e.g.], [ 42]–[44], the mechanism of enhanced scene encoding at target events is unknown. According to the theory of attentional boost effect [17], there may be greater activation not only in the attentional spotlight but also in peripheral locations at behaviorally relevant points in time [16]. When participants suppress distractors, this peripheral enhancement may be inhibited. We suggest that both peripheral enhancement, associated with focal attention to targets, and peripheral-inhibition, associated with focal suppression of distractors are deficient in PTSD. Future studies will explore these assumptions and their relationship with attentional alterations in the case of trauma-related and emotional cues [e.g.], [ 45]–[49], and the possible implications of these findings for the cognitive models of PTSD [9], [29], [45], [49], [50].
